# Supplementary material for: A Deep Learning Algorithm for Detecting Acute Pericarditis by Electrocardiogram
Source: J Pers Med. 2022 Jul 15;12(7):1150. doi: 10.3390/jpm12071150 (PMC9324403; doi:10.3390/jpm12071150)
Supplement: Supplementary file 1 [file jpm-12-01150-s001.zip › Supplementary Case S1-S5.pdf]

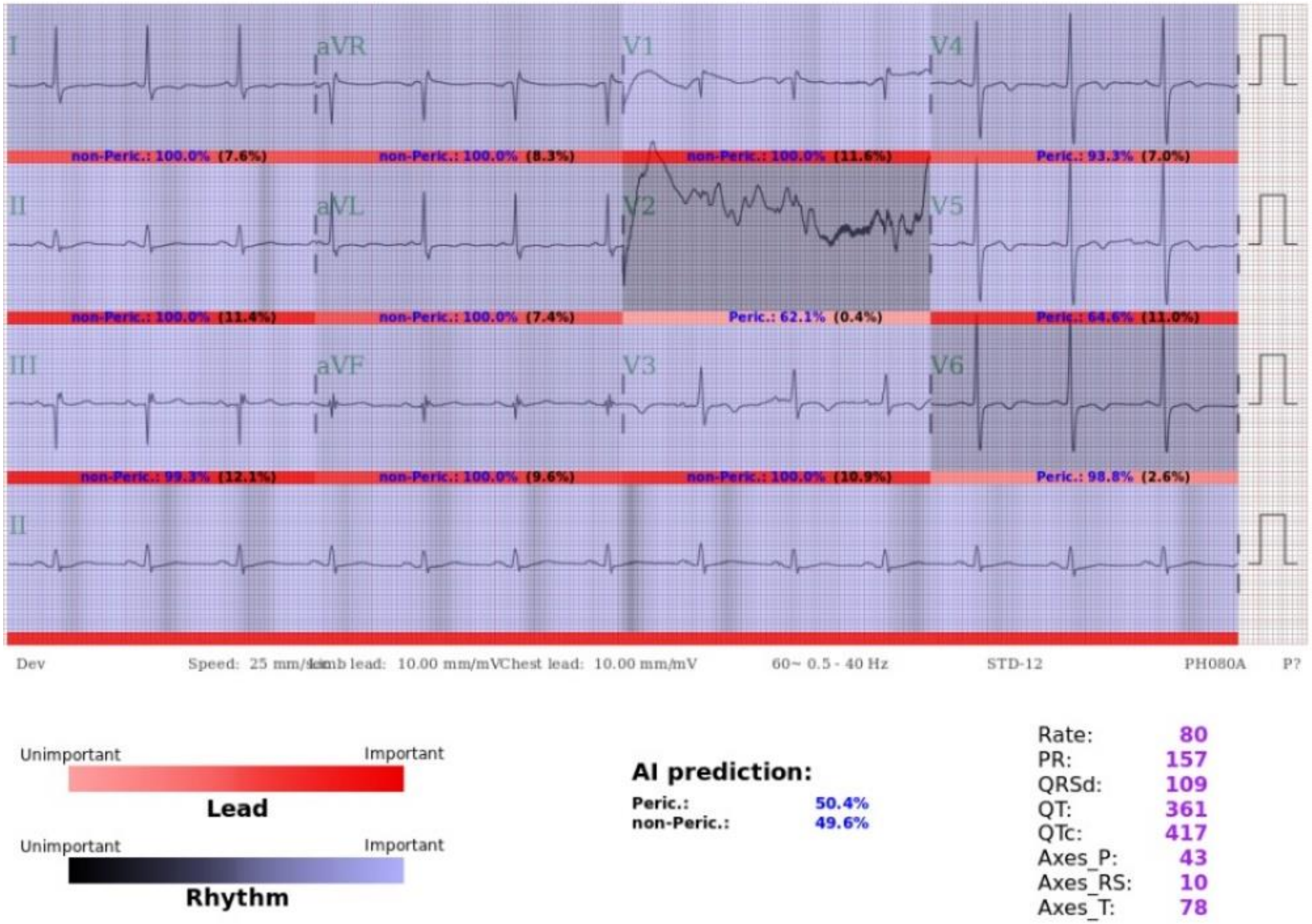

Supplementary Case S1. ECG of an acute pericarditis case identified by the pericarditis-DLM. A 77-year-old man presented with fever, acute chest pain and dyspnea. The ECG revealed sinus rhythm with inverted T waves in precordial leads and Q waves in leads III and aVF. Our pericarditis-DLM correctly recognized it as acute pericarditis, which was misdiagnosed by human experts.

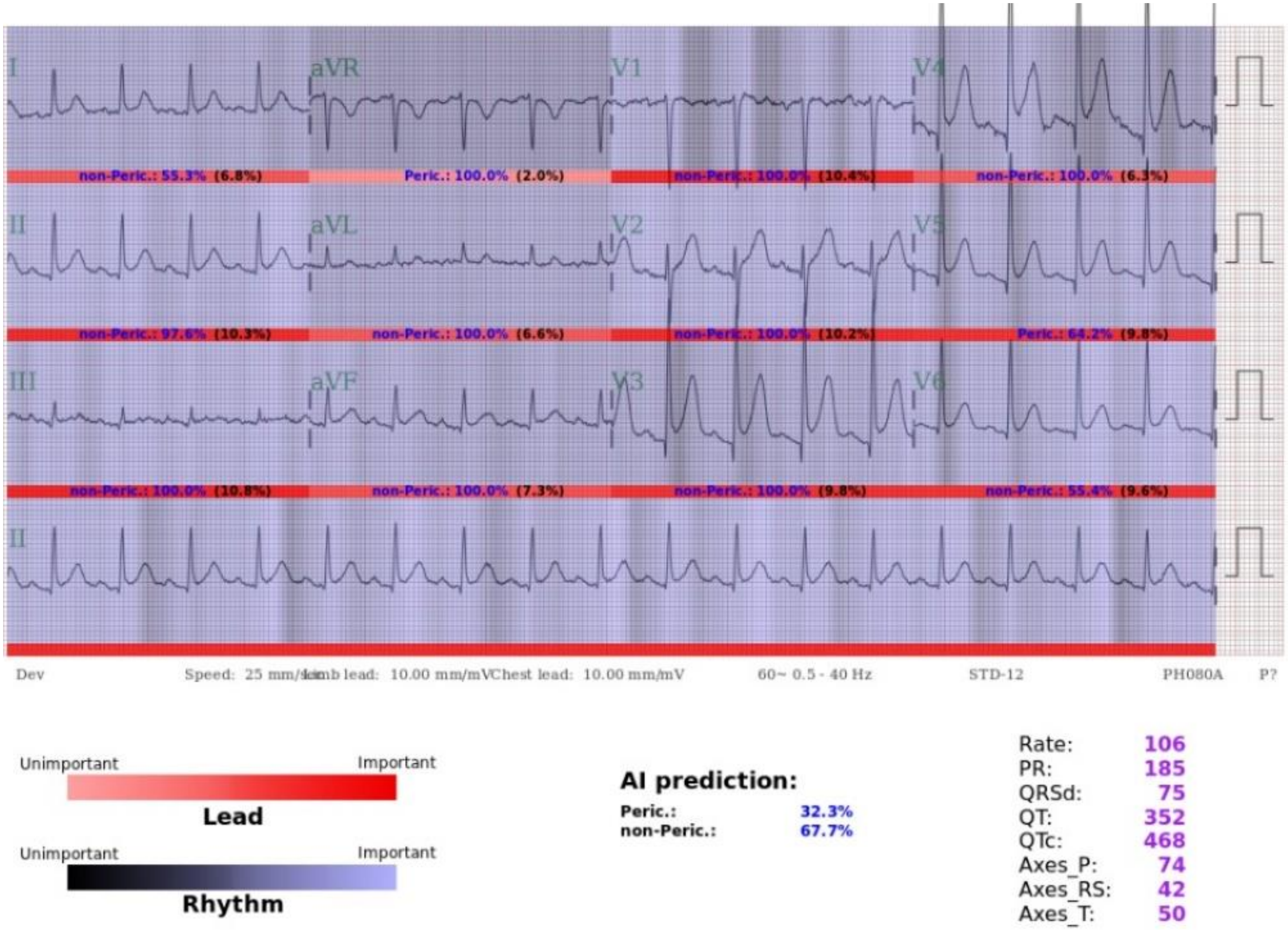

Supplementary Case S2. ECG of an acute pericarditis case but unrecognized by the pericarditis DLM. A 74-year-old man presented with fever, dyspnea, and sustained tachycardia. The ECG revealed sinus rhythm with widespread, concave ST-segment elevation and PR-segment elevation in lead aVR. Our pericarditis-DLM misidentified it as non-pericarditis, and all human experts correctly diagnosed it as acute pericarditis.
