# Supplementary material for: A Deep Learning Algorithm for Detecting Acute Pericarditis by Electrocardiogram
Source: J Pers Med. 2022 Jul 15;12(7):1150. doi: 10.3390/jpm12071150 (PMC9324403; doi:10.3390/jpm12071150)
Supplement: Supplementary file 1 [file jpm-12-01150-s001.zip › Supplementary Figure S1-S5.pdf]

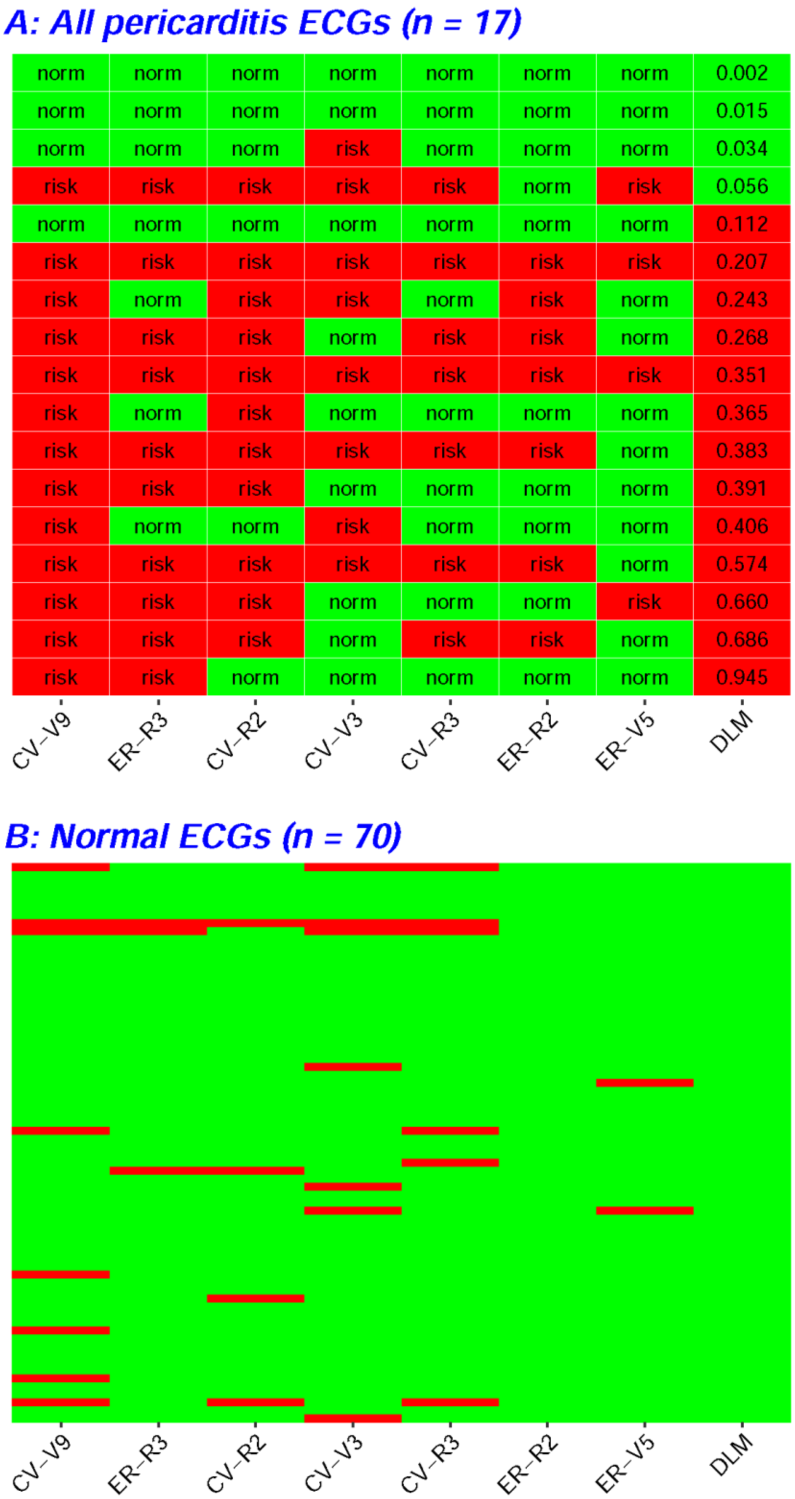

Supplementary Figure S1. Consistency analysis of answers given by the deep learning model and human experts. The answers given by our physicians were "risk" and "norm" corresponding to potential pericarditis and non-pericarditis ECGs, respectively. The numbers shown in the last column are the probability given by AI. The positive cut of points is 0.110 in this analysis based on Youden's index in the tuning set, and the colors green and red represent the low and high risk identified by AI, respectively.

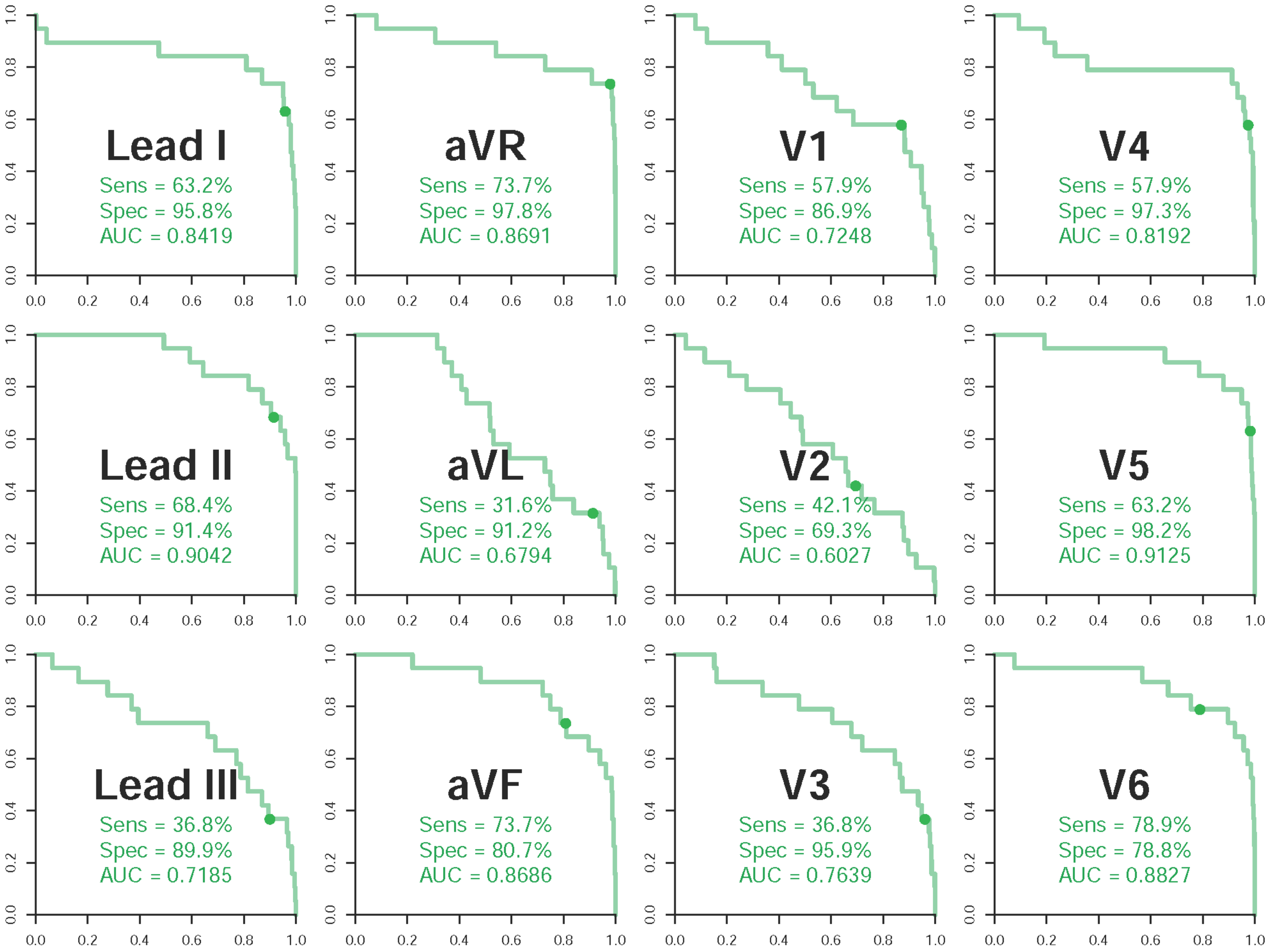

Supplementary Figure S2. Lead-specific performance analysis of pericarditis in the validation set. These are ROC curves with the specificity of the x-axis and the sensitivity of the y-axis. The cut points of the deep learning model were based on Youden's index in the tuning set.

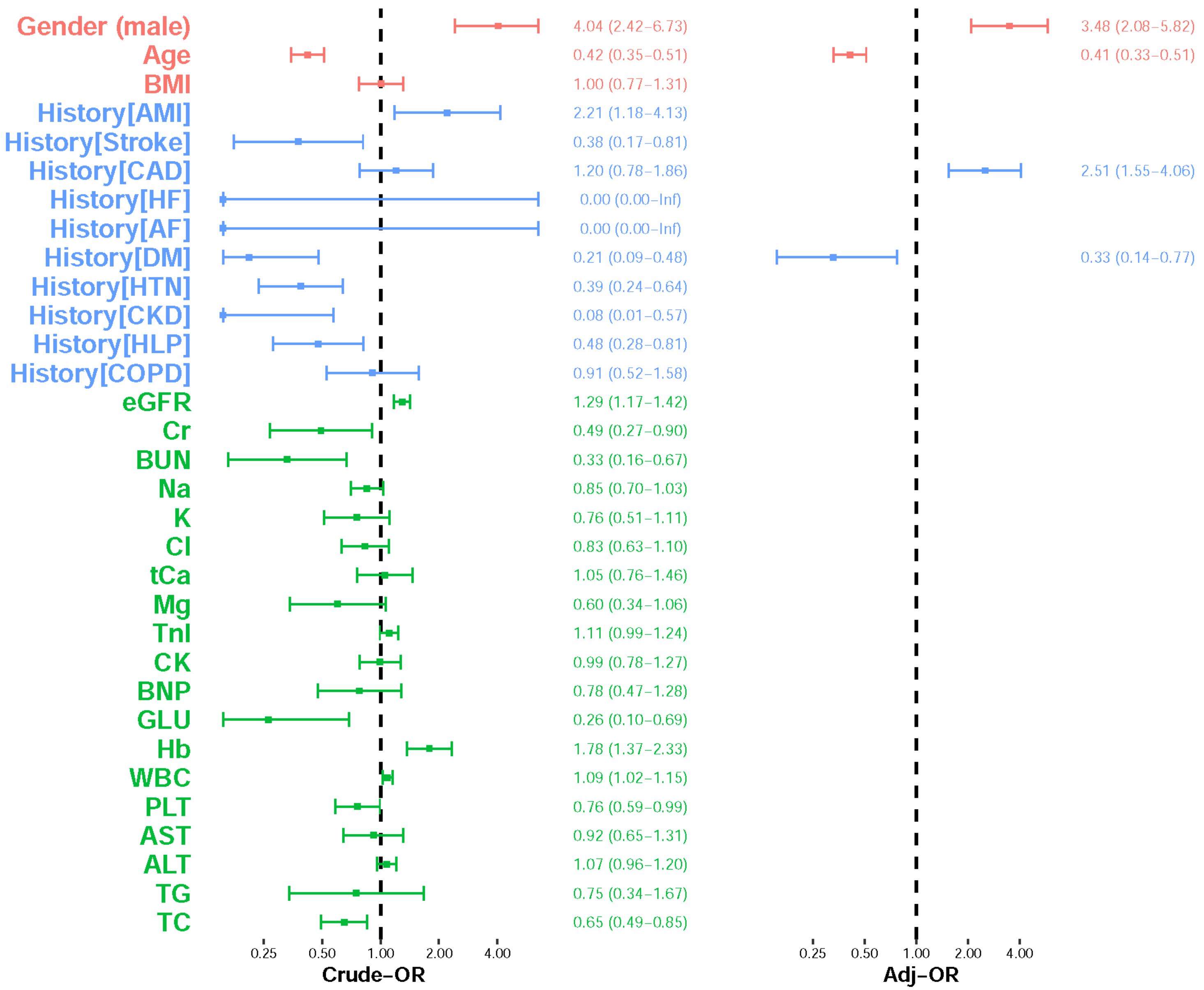

Supplementary Figure S3. Logistic regression analysis of pericarditis in the development set. The continuous variables are standardized by mean and standard deviation, so the units of each continuous variable were 1 standard deviation. The multivariable logistic regression analysis only included the significant variables.

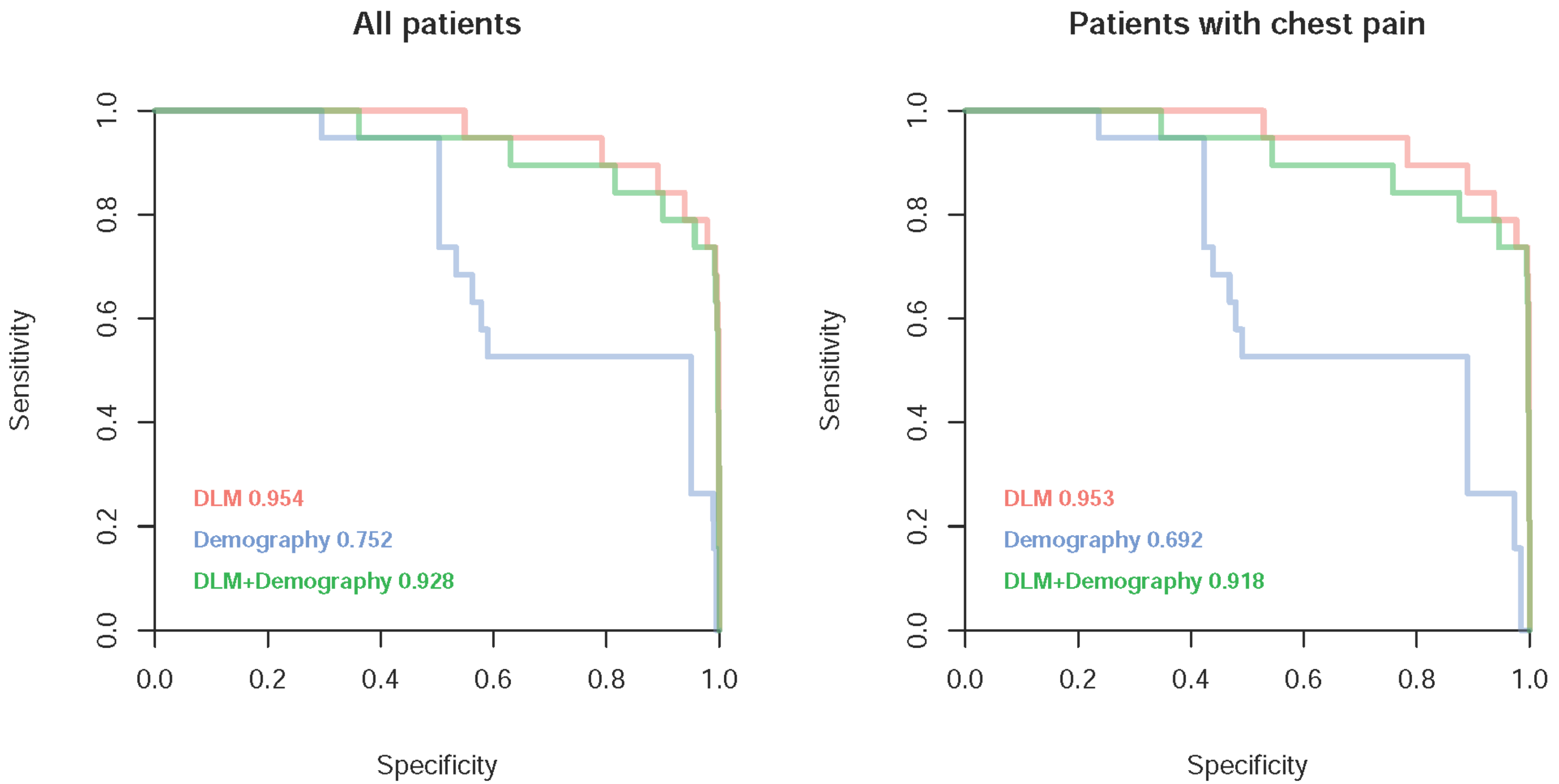

Supplementary Figure S4. Comparison of the diagnostic value between additional demographic variables and the deep learning model (DLM) in the validation set. The ROC curves were generated from the logistic regression analysis using the tuning set. Patient demographic variables included sex, age, CAD, and DM, which were significant in multivariable analysis, as shown in Figure S3.

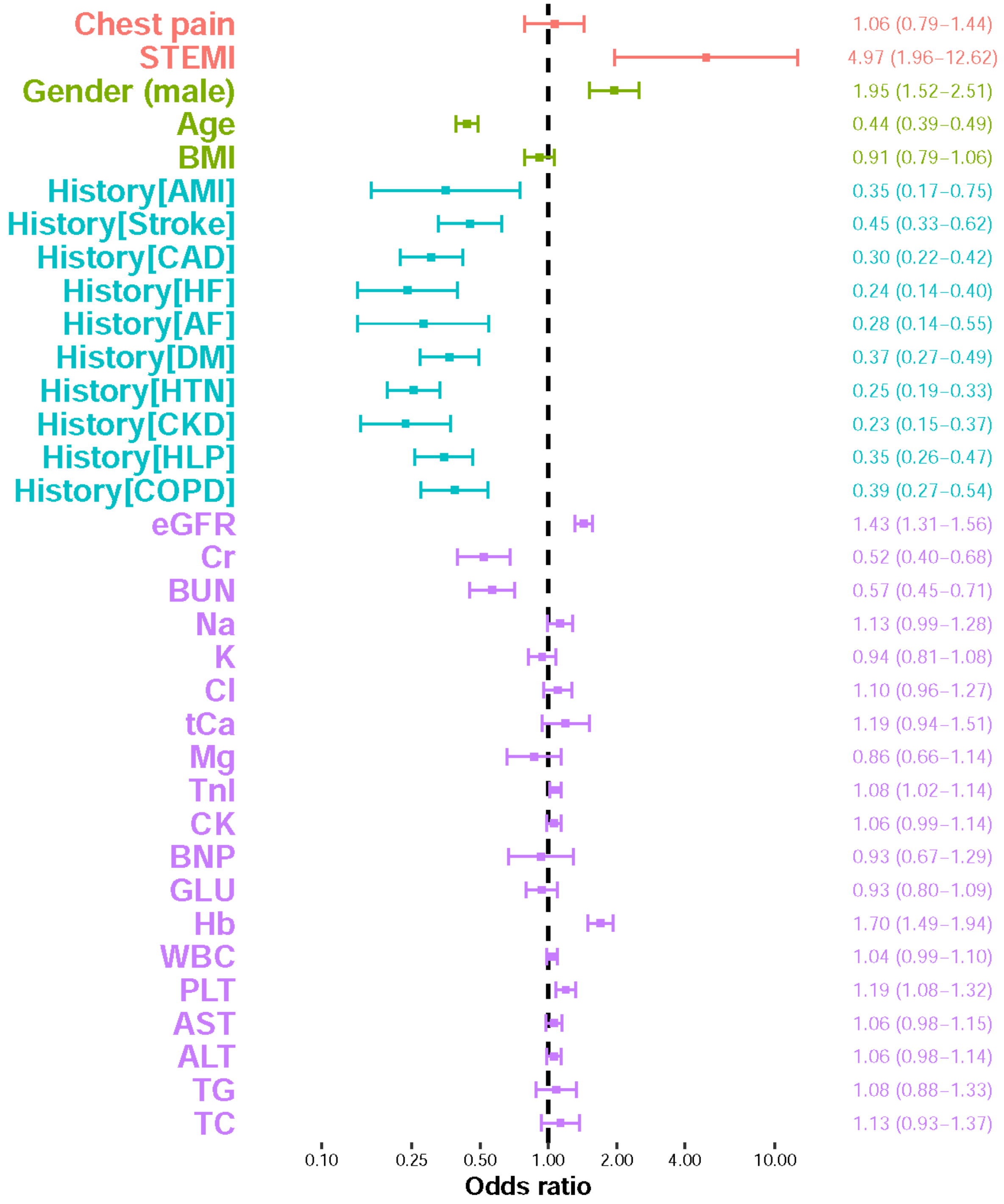

Supplementary Figure S5. Misclassification analysis of non-pericarditis in the validation set. The positive samples in this analysis were non-pericarditis patients with a DLM-defined abnormal ECG (n = 294), and the negative samples were non-pericarditis patients with a DLM-defined normal ECG (n = 12,752). The continuous variables are standardized by mean and standard deviation, so the units of each continuous variable were 1 standard deviation.

Protect pdf from copying with Online-PDF-No-Copy.com
